# Supplementary figures and images for: KSHV infection of endothelial precursor cells with lymphatic characteristics as a novel model for translational Kaposi’s sarcoma studies
Source: PLoS Pathog. 2023 Jan 23;19(1):e1010753. doi: 10.1371/journal.ppat.1010753 (PMC9894539; doi:10.1371/journal.ppat.1010753)

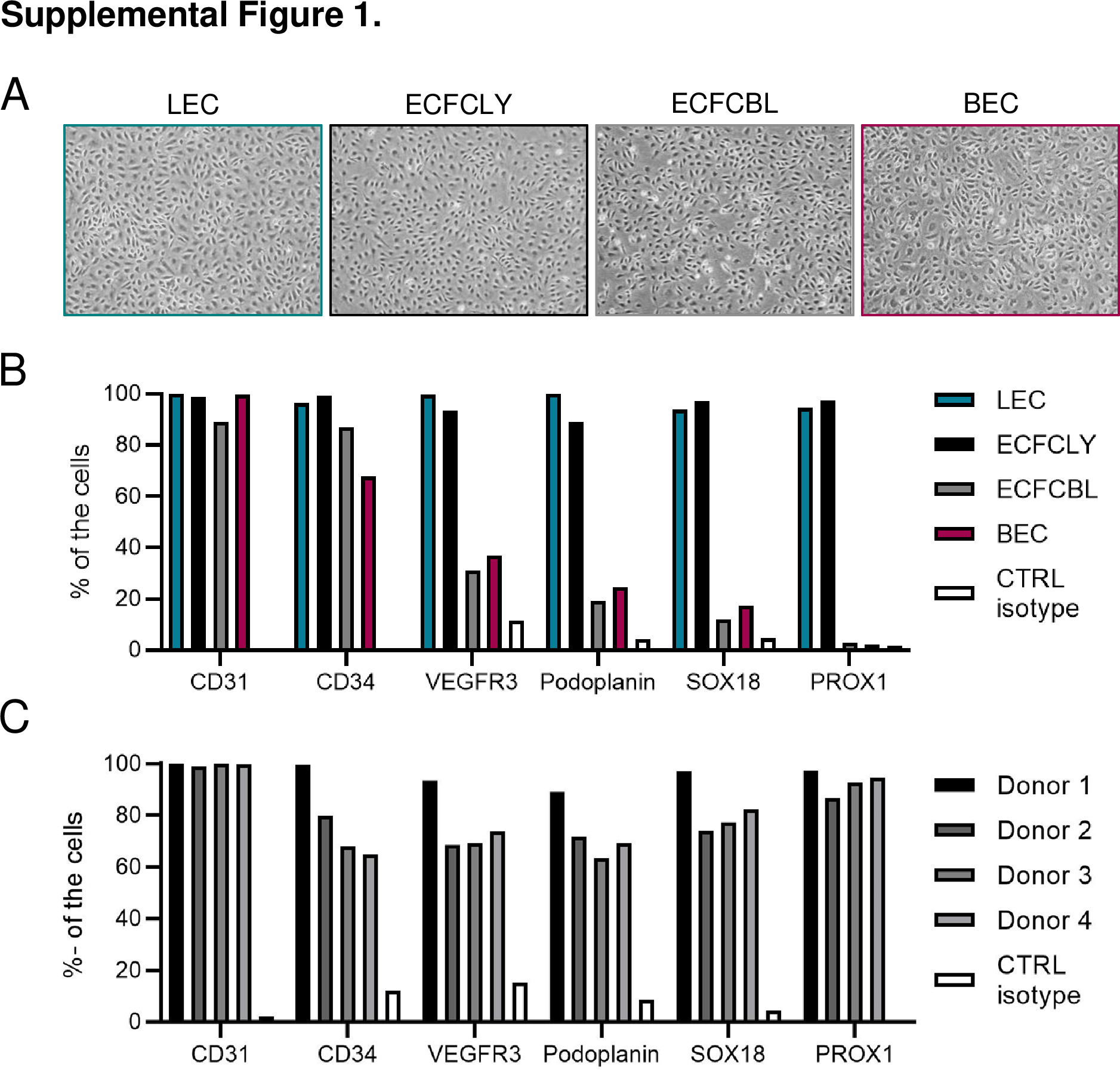

Supplement: S1 Fig — Comparison of the adherent ECFC isolates to the mature LECs and BECs. A. Phase contrast microscopic images. B. Expression of surface (CD31, CD34, VEGFR3, and Podoplanin) and nuclear (SOX18 and PROX1) endothelial cell markers analyzed by FACS. Corresponding IgG isotype antibodies were used as controls. C. The endothelial cell markers in B analysed by FACS from ECFCs isolated from four different healthy donors. (TIF) [file ppat.1010753.s001.tif]

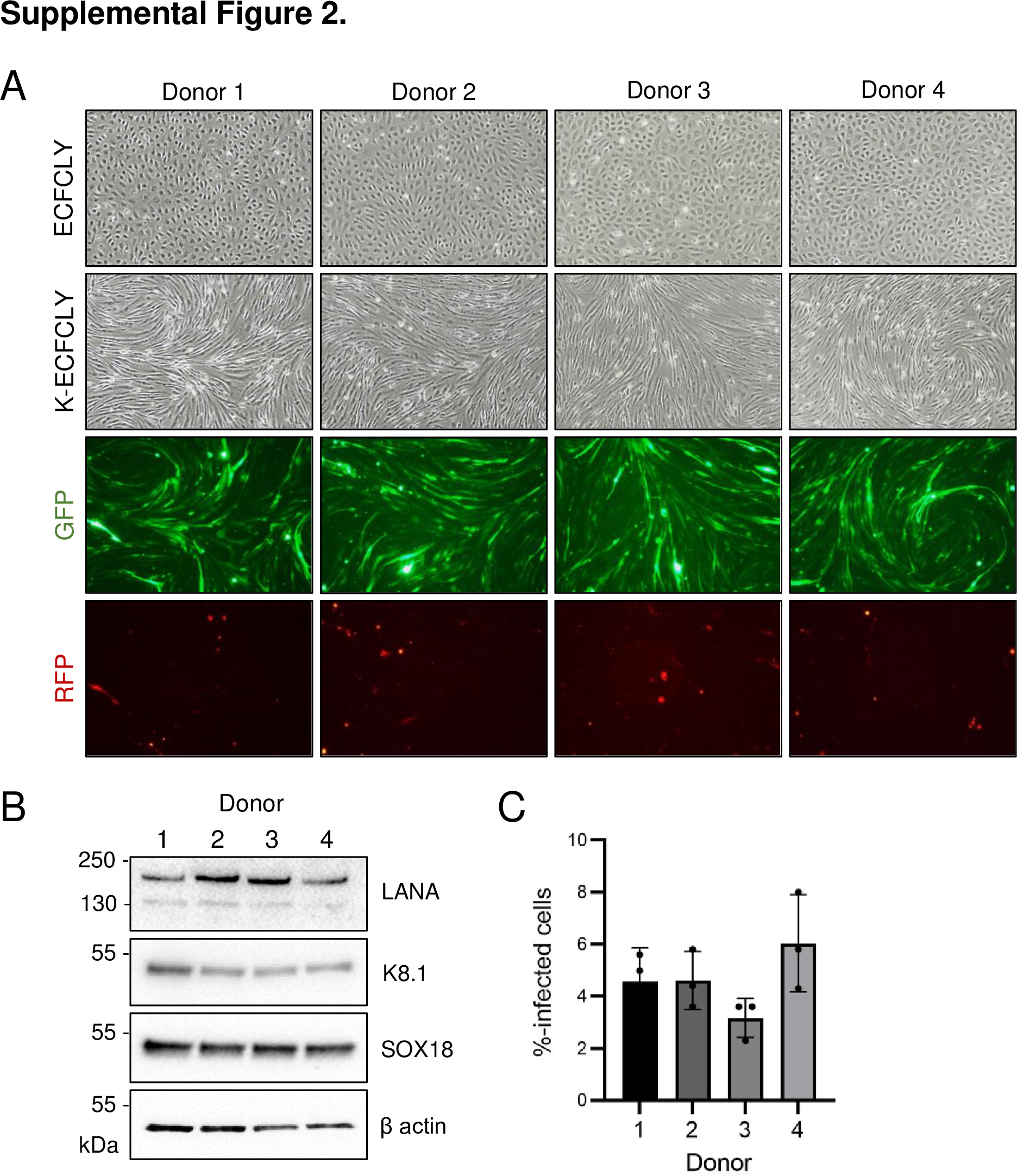

Supplement: S2 Fig — ECFCLYs, isolated from four different donors, were infected with rKSHV.219. A. Pictures taken at 7 d.p.i show spindling phenotype, latent infection (GFP) and spontaneous lytic replication (indicated by RFP expression). B. Expression of KSHV latent (LANA) and lytic (K8.1) proteins and SOX18. C. KSHV titers were measured from virus release assay on naïve U2OS cells. (TIF) [file ppat.1010753.s002.tif]

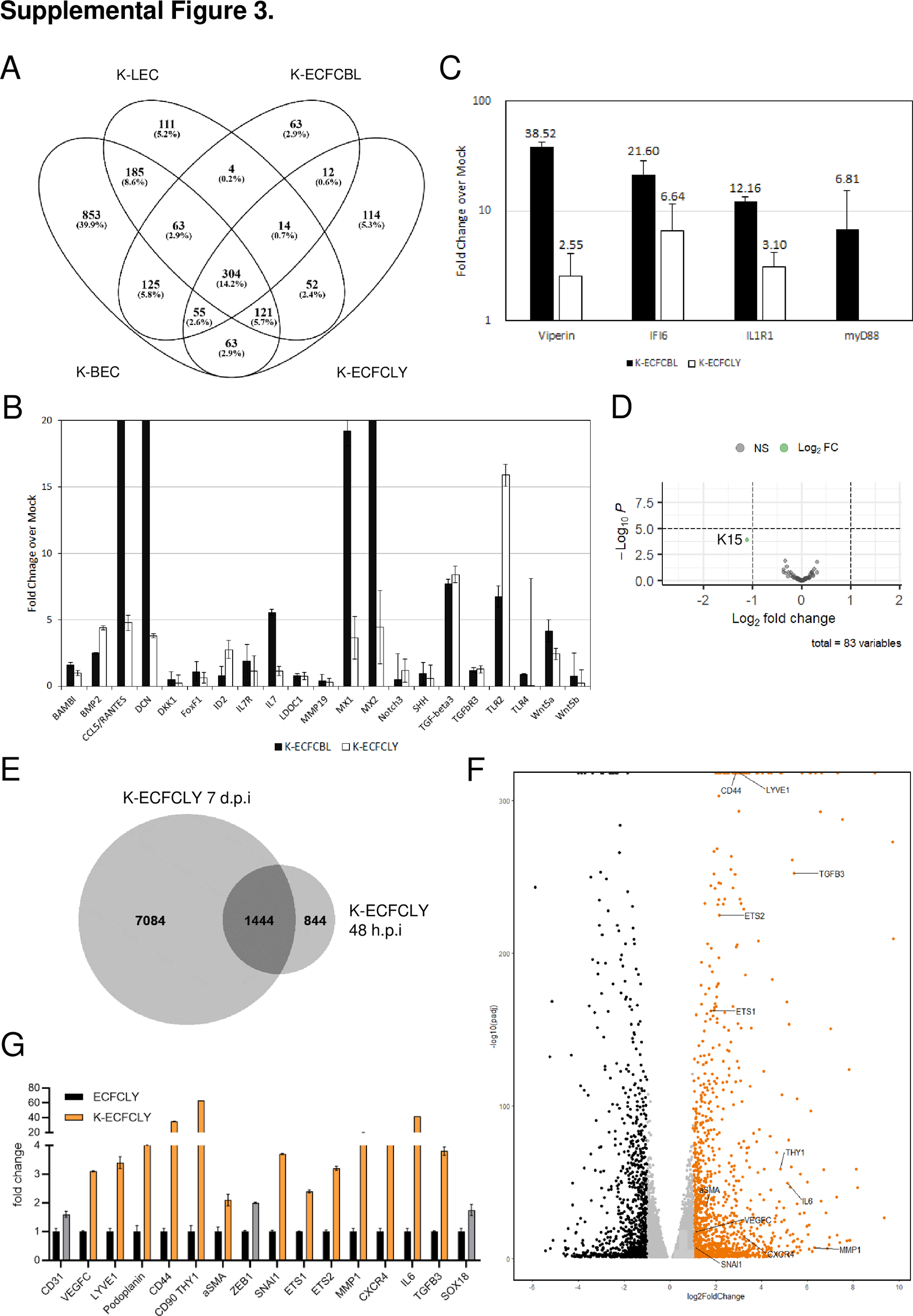

Supplement: S3 Fig — A. Venn diagram showing overlapping gene expression profiles of wtKSHV-infected BEC and LEC along with blood and lymphatic ECFCs. B and C. Blood (black bars) and lymphatic (white bars) ECFCs were mock- or KSHV-infected. At 48 h.p.i, RNA was isolated and analyzed for gene expression of (B) a selection of genes and of (C) immune response genes identified as changed by RNA-sequencing. D. Volcano plot of K-ECFCBL and K-ECFCLY reads aligned to the KSHV genome indicating only one gene, K15, differentially expressed between the two cell types. E-G. Lymphatic ECFCs were mock or rKSHV.219-infected, and 7 d.p.i RNA was isolated and analyzed for gene expression by RNA-sequencing. Common gene expression changes between different isolates of ECFCLY after KSHV-infection are shown as Venn diagram (E). Volcano blot with at least 2-fold up- (orange) and downregulated (black) genes with adjusted P value < 0.05 (F), and validation of selection of genes by qPCR (G). RNA-sequencing was done with biological triplicates of each sample. (TIF) [file ppat.1010753.s003.tif]

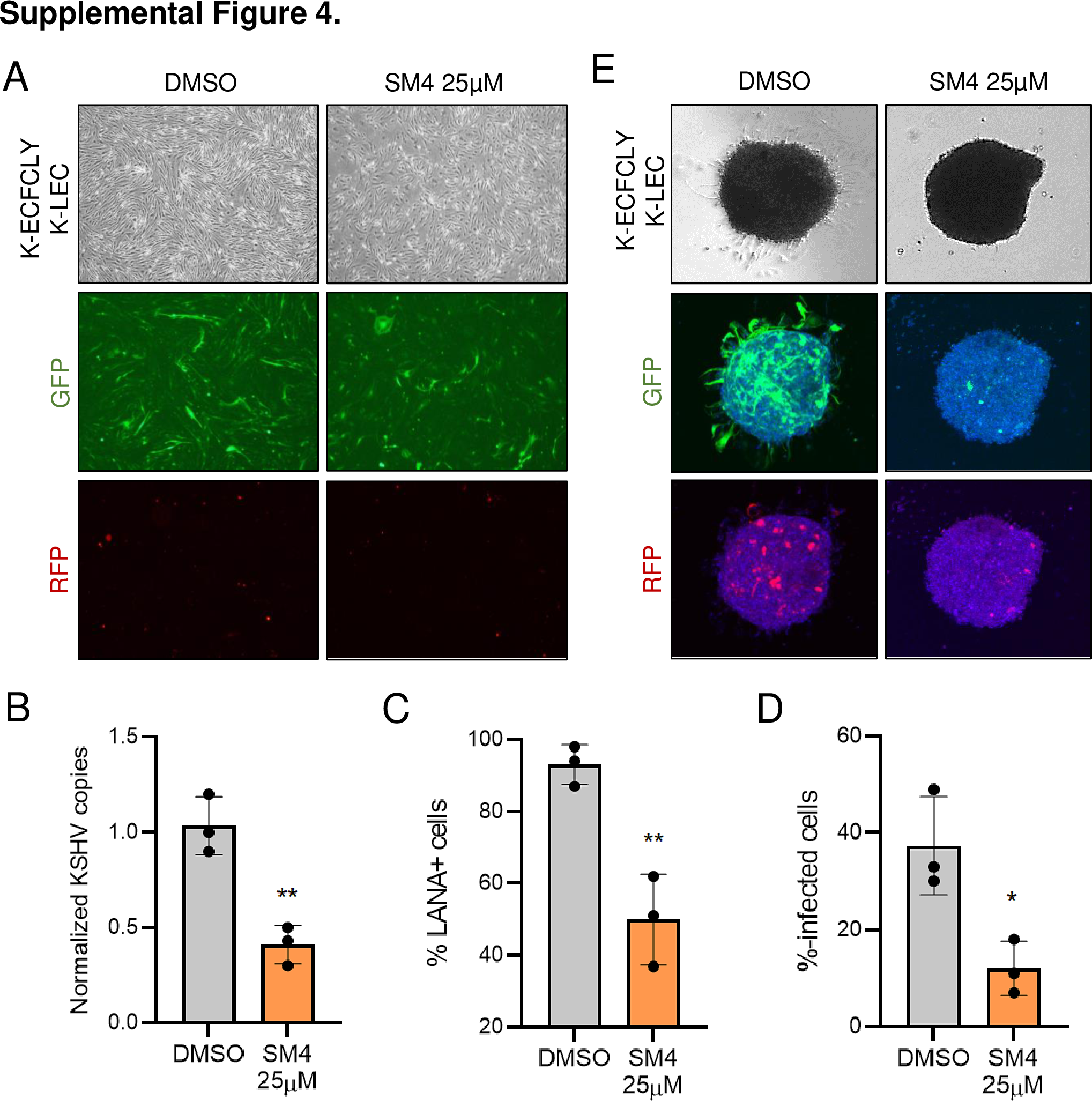

Supplement: S4 Fig — Lymphatic ECFCs and LECs were mixed at a ratio of 95% to 5% and infected with KSHV for five days. A. Cells were treated with 25 μM SM4 or DMSO control for six days, replenished at day three. The effect of the treatments is shown as quantification of KSHV genome copies from total DNA (B), percentage of LANA positive cells on 96-well plates (C) and KSHV titers measured by the virus release assay on naïve U2OS cells (D). E. Infected ECFC/LEC mixture was allowed to form spheroids overnight and then embedded in 3D fibrin. Spheroids were treated for six days with either 25 μM SM4 or DMSO control, fixed and imaged. Phase contrast and confocal images are shown at treatment day six. * p < 0.05, ** p < 0.01. (TIF) [file ppat.1010753.s004.tif]

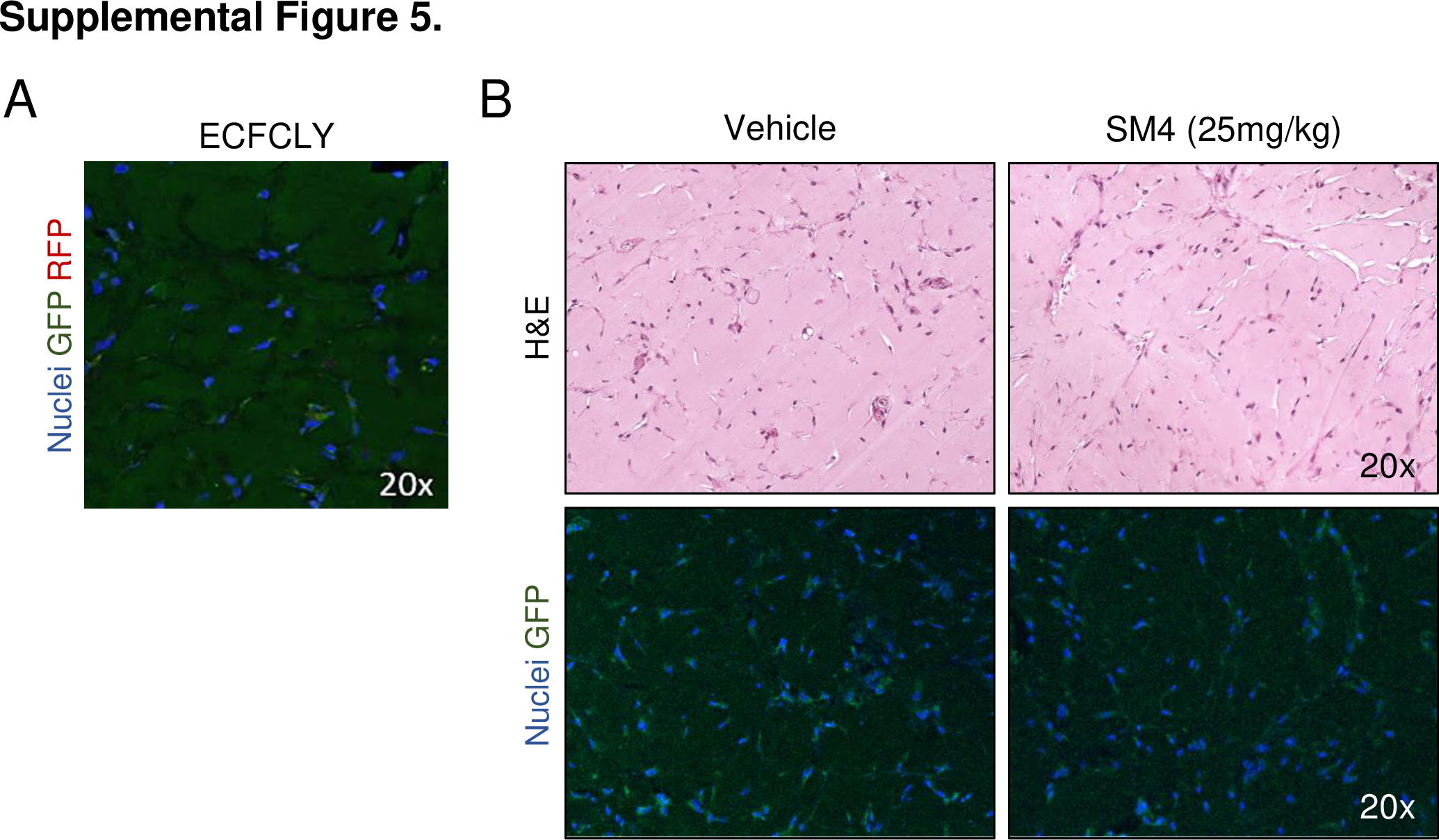

Supplement: S5 Fig — Uninfected ECFCLYs were implanted subcutaneously into NSG mice and collected 30 days later (A) or after a 10-day treatment with either Vehicle or SM4 (25 mg/kg) (B) for histological analyses by IHC and IF. (TIF) [file ppat.1010753.s005.tif]
