# Supplementary material for: KSHV infection of endothelial precursor cells with lymphatic characteristics as a novel model for translational Kaposi’s sarcoma studies
Source: PLoS Pathog. 2023 Jan 23;19(1):e1010753. doi: 10.1371/journal.ppat.1010753 (PMC9894539; doi:10.1371/journal.ppat.1010753)
Supplement: S1 Text — (DOCX) [file ppat.1010753.s006.docx]

**Supplemental Text 1.**

**Table A. Gene Ontology categories enriched in KSHV-infected blood ECFCs.**

| **GO Term** | **Adjusted P-value** |
| --- | --- |
| response to other organism | 1.56E-04 |
| response to biotic stimulus | 2.53E-04 |
| immune system process | 1.17E-03 |
| response to lipopolysaccharide | 1.17E-03 |
| immune response | 1.49E-03 |
| response to molecule of bacterial origin | 1.49E-03 |
| response to virus | 2.91E-03 |
| multi-organism process | 6.01E-03 |
| response to fluid shear stress | 1.08E-02 |
| coagulation | 1.86E-02 |

**Table B. Gene Ontology category genes enriched in KSHV-infected blood ECFCs.**

| **GO Term** | **Genes** |
| --- | --- |
| response to other organism | TLR2 HERC5 RSAD2 SAMHD1 IDO1 IL6R DCN CCL5 PLSCR1 THBD ISG15 F3 TPO MX1 MX2 |
| immune response | IL1R1 CXCL5 NCF2 SMAD6 CXCL2 TLR2 HERC5 SAMHD1 RSAD2 OAS1 IFI44L OAS2 IL6R CCL5 MYD88 SEMA7A IFI6 |
| response to virus | PLSCR1 ISG15 HERC5 SAMHD1 RSAD2 CCL5 MX1 MX2 |
