# Supplementary material for: KSHV infection of endothelial precursor cells with lymphatic characteristics as a novel model for translational Kaposi’s sarcoma studies
Source: PLoS Pathog. 2023 Jan 23;19(1):e1010753. doi: 10.1371/journal.ppat.1010753 (PMC9894539; doi:10.1371/journal.ppat.1010753)
Supplement: S2 Text — (DOCX) [file ppat.1010753.s007.docx]

**Supplemental Text 2.**

**Table A. Gene Ontology categories enriched in KSHV-infected lymphatic ECFCs.**

| **GO Term** | **Adjusted P-value** |
| --- | --- |
| negative regulation of multicellular organismal metabolic process | 5.25E-03 |
| negative regulation of collagen metabolic process | 5.25E-03 |
| lymph vessel development | 1.92E-02 |
| intracellular signal transduction | 1.99E-02 |
| intracellular protein kinase cascade | 2.36E-02 |
| signal transduction | 2.36E-02 |
| signal transmission via phosphorylation event | 2.36E-02 |
| cardiac epithelial to mesenchymal transition | 3.42E-02 |
| intracellular signaling pathway | 3.42E-02 |
| signaling | 3.42E-02 |

**Table B. Gene Ontology category genes enriched in KSHV-infected lymphatic ECFCs.**

| **GO Term** | **Genes** |
| --- | --- |
| negative regulation of collagen metabolic process | CIITA CST3 HGF |
| lymph vessel development | PDPN EFNB2 SOX18 |
| signal transduction | RHOJ ADCY1 RAB3C PPP2R5A ITPKA RHOU GPR3 TRIB1 STAT4 GCKR RASGRP3 UNC5B OSR1 DGKD LTB4R MAP3K8 SPRED3 INSR BMP2 SPARCL1 PDPN PIK3C2A PDK4 HGF MAP4K4 PDE7B ERBB2IP CD33 HIPK2 TGFBR3 PPM1L NRGN TIAF1 |
| cardiac epithelial to mesenchymal transition | BMP2 TGFBR3 |
