# Supplementary material for: KSHV infection of endothelial precursor cells with lymphatic characteristics as a novel model for translational Kaposi’s sarcoma studies
Source: PLoS Pathog. 2023 Jan 23;19(1):e1010753. doi: 10.1371/journal.ppat.1010753 (PMC9894539; doi:10.1371/journal.ppat.1010753)
Supplement: S3 Text — (DOCX) [file ppat.1010753.s008.docx]

**Supplemental Text 3.**

| **Enriched gene sets in KSHV-infected LEC** |  |
| --- | --- |
| **Gene Set Name** | **p-value** |
| HALLMARK_TNFA_SIGNALING_VIA_NFKB | 4.96E-18 |
| HALLMARK_IL2_STAT5_SIGNALING | 9.76E-13 |
| HALLMARK_HYPOXIA | 9.78E-11 |
| HALLMARK_KRAS_SIGNALING_UP | 6.93E-09 |
| HALLMARK_IL6_JAK_STAT3_SIGNALING | 4.14E-08 |
| HALLMARK_INFLAMMATORY_RESPONSE | 3.69E-07 |
| HALLMARK_INTERFERON_GAMMA_RESPONSE | 3.69E-07 |
| HALLMARK_COMPLEMENT | 2.40E-06 |
| HALLMARK_P53_PATHWAY | 2.40E-06 |
| HALLMARK_ADIPOGENESIS | 1.43E-05 |
|  |  |
| **Enriched gene sets in KSHV-infected ECFCLY** |  |
| **Gene Set Name** | **p-value** |
| HALLMARK_TNFA_SIGNALING_VIA_NFKB | 2.18E-19 |
| HALLMARK_IL2_STAT5_SIGNALING | 8.82E-14 |
| HALLMARK_INTERFERON_GAMMA_RESPONSE | 9.66E-14 |
| HALLMARK_APOPTOSIS | 5.89E-09 |
| HALLMARK_COMPLEMENT | 9.66E-09 |
| HALLMARK_INFLAMMATORY_RESPONSE | 9.66E-09 |
| HALLMARK_KRAS_SIGNALING_UP | 9.66E-09 |
| HALLMARK_HYPOXIA | 7.81E-08 |
| HALLMARK_P53_PATHWAY | 7.81E-08 |
| HALLMARK_IL6_JAK_STAT3_SIGNALING | 1.58E-07 |
