# Supplementary material for: KSHV infection of endothelial precursor cells with lymphatic characteristics as a novel model for translational Kaposi’s sarcoma studies
Source: PLoS Pathog. 2023 Jan 23;19(1):e1010753. doi: 10.1371/journal.ppat.1010753 (PMC9894539; doi:10.1371/journal.ppat.1010753)
Supplement: Attachment — Submitted filename: Response to Reviewers.pdf [file ppat.1010753.s011.pdf]

PLoS Pathogens

KSHV infection of endothelial precursor cells with lymphatic characteristics as a novel model for translational Kaposi's sarcoma studies

D-22-01283

Part I - Summary

Please use this section to discuss **strengths/weaknesses** of study, **novelty/significance**, **general execution and scholarship**.

Reviewer #1:

Kaposi Sarcoma is a malignancy characterized by hyper-angiogenesis and highly proliferative spindle-shaped cells infected with Kaposi Sarcoma Herpesvirus (KSHV). The origin of these spindle cells has been elusive. Candidates include blood endothelial cells, lymphatic endothelial cells and mesenchymal stem cells. Previous work by this group indicated the presence of a circulating endothelial cell that harbored KSHV. Here the authors examine if circulating endothelial cell precursors termed endothelial colony forming cells (ECFCs) are permissive for KSHV infection and recapitulate aspects of KS. Major findings of their descriptive report are that: (1) lymphatic ECFCs were more permissive to KSHV and maintain viral episomes better than blood ECFCs; (2) infection of KSHV resulted in growth inhibition under 2D culture but increased survival in 3D culture; (3) and that infection has differential impacts on the host gene expression profile in lymphatic and blood ECFCs. They also confirm previous findings that Sox18 is a key host factor for lymphatic ECFCs to support KSHV infection. In summary, this well-written manuscript reports an exciting new primary cell type that is susceptible to infection and that may inform reservoirs of KSHV infection and pathogenesis in individuals. However, their characterization is superficial, and it is unclear how these precursor cells compare to LECs and infected ECs in skin KS tumors.

Reviewer #2:

This manuscript describes studies designed to identify the origin of the KS spindle cell, thus addressing an extremely important but unresolved question. The focus is on lymphatic endothelial cells (LEC), and more specifically on circulating endothelial colony forming cells (ECFCs) that express markers of lymphatic, not blood, endothelium. The premise that KSHV-infected lymphatic ECFCs are indeed the spindle cell precursors is based on a sound body of previous work contributed by the laboratories of the two corresponding authors, and by several independent studies. Additional benefits of this work include a comparison of KSHV infection dynamics in blood ECFCs and a demonstration that lymphatic ECFCs can be used in a mouse model for in vivo KS/KSHV translational studies. Overall, this is a well-performed and accurately reported body of work. However, a few additional experiments as well as minor clarifications and details are requested. For example, since an important component of this work's contribution is method development, certain methods and materials should be described in more detail. In addition, the rationale for certain comparisons and the significance of the reported outcomes is not always clearly or sufficiently articulated. Finally, while I acknowledge the authors' recognition that this work is in part intended to validate further characterization of the ECFC model in the future, there are a few additional experiments that would really benefit this particular study, in particular more work with sprouting assays and an in vitro validation of the cell mixing protocols used in the in vivo model.

Reviewer #3:

KS spindle cells are known to be of endothelial origin, but it is not certain whether blood endothelial cells (BECs) or lymphatic endothelial cells (LECs) are the source. Another possibility is that KSHV infects circulating endothelial precursors known as endothelial colony forming cells (ECFCs). ECFCs themselves can be divided into blood and lymphatic subtypes. In this work the authors sought to better clarify which cell type ultimately forms spindle cells in KS tumors. They found that lymphatic ECFCs, much like LECs, were more permissive to KSHV infection, maintained the viral episome longer, and produced more infectious virus than blood ECFCs or BECs. Lymphatic ECFCs also supported spontaneous lytic reactivation. In addition, it was determined that KSHV-infected lymphatic ECFCs, but not infected blood ECFCs or uninfected cells, were able to form small colonies in soft agar. Importantly, the authors were able to use lymphatic ECFCs to develop an in vivo model of KS and use it to test the efficacy of SM4 as a potential treatment. This work not only addresses a significant question in the KSHV field but provides a promising in vivo model which can be used to assess future drug candidates for the treatment of KS.

Part II – Major Issues: Key Experiments Required for Acceptance
Please use this section to detail the key new experiments or modifications of existing experiments that should be absolutely required to validate study conclusions.

**Reviewer #1**:	**Response**
1. The status of KSHV infection was inferred nearly exclusively by reporter fluorescent proteins that do not reflect viral gene expression programs. Staining of LANA as a marker of latency and K8.1 (or other lytic protein) as lytic makers are suggested to give more clarity regarding the % of cells in either program.	Figures 1 A, B and F previously showed quantification of LANA+ staining and therefore used LANA as a marker of latency. We have added additional demonstration of latent and lytic infection by showing immunofluorescence staining of LANA and K8.1 in Figure 1 panel C. The description is also added to the methods, page 27, lines 582-591).
While host DEGS are defined based on RNAseq data, there is no comprehensive examination of viral gene expression in the K-ECFCLYs. It is difficult to understand the kinetics and lifecycle of KSHV in these cells without a better analysis.	As requested, we mapped reads from our original RNAseq data of infected K-ECFCLY and K-ECFCBL to the viral genome to identify comprehensive expression of viral genes. The comparison is described in the results section and shown in Supplemental Figure 3, panel D.
2. In Figure 1, more controls are needed to better compare different cell types. Pictures taken with KSHV infected ECFCBL and uninfected cells are missing in Figure 1C, in Figure 1E and Figure 1G, the counterpart with K-ECFCBL is also missing. In Figure 1D, the WB for indicated proteins in ECFCBL and K-ECFCBL would be useful.	To respond to the reviewer's requests, additional cell culture work for Figures 1D, E, F and H was carried out in Finland using ECFCBL cells sent from the Lagunoff lab in Seattle, USA and compared to the ECFCLY from Finland. The data from these additional experiments are now included in the revised Figure 1, panels C-F and H.<br><br>Images of uninfected cells of each type are shown in the Supplemental Figure 1 panel A, and thus were not included in the main Figure 1. A picture of the ECFCBL culture and data from a FACS analysis have now also been added to S1 Fig.
3. In Figure 2, how would the authors explain infection of KSHV slowed down infected cell growth at early time point (60hpi) in Figure 2A but had little effect when examined at late time point (5dpi) in Figure 2B?	The differences are at least partially due to differences in the data acquisition rather than the timepoint. In Figure 2A, we are measuring confluence, which does not take into account any increased cell death, which we know occurs in KSHV-infected samples. In contrast, Figure 2B is measuring changes to proliferation specifically, which we have shown to not be changed in KSHV-infected LECs.
In Figure 2C, it seems that KSHV might be inducing autocrine growth factors. Did the authors examine for secreted factors that drive survival in the conditioned media of the growth factor reduced conditions.	Thank you for bringing up this interesting point. However, we did not study whether infected cells secreted factors promoting survival as this was not within the scope of our study.
Would different time points post-infection be a factor in determining the outcome of the tube formation assay in 2D?	While it is formally possible that later times post-infection may have effects on capillary-like tube formation, we chose to perform these experiments at 24 hpi because latency has been established and we were asking whether latent KSHV infection affects tube formation and stability as we have previously observed differences in tube stability in latent KSHV infection of primary BECs but not LECs at this time point *(DiMaio et al. 2014, PMID: 25275137)*.

4. Figure 3A, while there were colonies observed with K-ECFCLY on day 36, the total cell number in the field was even lower than that with control ECFCLY. How do the authors distinguish colony formation from mere clustering?	We carefully examined the cells after embedding them in the agarose. As stated, we noted no clustering of cells after plating. As the cells were embedded in a semisolid agarose matrix where they are unable to migrate and cluster, the only cluster formed could be from cell proliferation.
Could the authors better elaborate the difference between experiments in 3A and 3C where colonies seem improved?	As described in the manuscript, the cells in panel A were isolated by different means than the cells in panel C. While in both cases only the lymphatic ECFCs formed small clusters, the size of the clusters vary between experiments. Importantly, in panel A, the cells were infected with wtKSHV for 48h before their embedding into soft agar, whereas in panel C, the cells were infected with rKSHV.219 for 5 days before embedding. Hence, longer infection time prior to embedding may improve the efficiency of colony formation. A more detailed description of the difference in the experimental conditions is now added to the manuscript text (page 31, lines 655-664).
For the donors 1-4, cells per colony were not provided for the uninfected cells.	For the donors 1-4 in Figure 3, panel E, quantification for the uninfected cells was not originally shown as no colonies formed when using uninfected cells. Only colonies with 3 or more cells in the images were included in the quantification. However, we agree that showing the uninfected counterparts strengthens the finding, and have therefore now included them (indicated as 'mock') to the revised panel E.
5. Supplemental Figure 3 would be better if incorporated into main Figures.	We appreciate the reviewer's interest in Supplemental Figure 3 being a main figure of the paper. However, while this figure provides additional information regarding the differences of the two ECFC cell types, it does not add to the main points of the paper. The main manuscript does not follow up these findings and therefore, we believe that this figure should remain as a supplementary figure showing the transcriptional differences in addition to the functional differences in infection.
Were there any DEGs that explain the nature of suppressed proliferation upon infection?	As shown in Supplemental Tables 1-4, we performed gene set enrichment to determine the classes of genes induced by KSHV in ECFCs. The top gene sets found to be altered were specifically involved in cell proliferation, however, these gene sets are quite broad and some are focused on general cell signaling pathways that may influence proliferation.
Is SOX18 expression upregulated in ECFCLY upon KSHV infection?	We thank the reviewer for this important question. SOX18 expression is indeed upregulated at mRNA level in ECFCLY upon KSHV infection (now added to the Supplemental Figure 3, as panel G), but much less than at protein level (Figure 1, panel D). SOX18 was not shown in the original Supplemental Figure 3 panels since its logarithmic fold change (FC) values were below the threshold that we had chosen for the significant differential gene expression changes in the RNAseq analysis.
They include a Venn diagram of LEC and ECFCLY with and without infection- does hierarchical analysis provide any insight regarding shifts in pathways caused by the virus that are common to both. Does	We have included a new Supplemental Table 5 showing enriched pathways in both K-LEC and K-ECFCLY cells compared to their corresponding mock cell type. Several of the pathways are enriched in both cell types when infected with KSHV. However, since there are no specific markers that differentiate ECFCLY and LEC it is

	KSHV infection drive the ECFCLY to a phenotype more like LECs?	difficult to determine whether KSHV drives the phenotype towards one of these cell types over the other.
6.	In Figure 4, the authors did not report cell toxicity of SM4 at the doses tested. Figure 4 F-H studies the dose-depending effect of SM4 on KSHV replication in ECFCLY, while Figure 4E showed the effect of SM4 at 25um while Figure 4I and 4J reported the effect of SM4 at 50um. Cells in the spheroid of SM4 treated K-ECFCLY appear unhealthy.	We agree that possible toxicity is very important to report when testing new potential treatment modalities. We have added a new panel F in Figure 4 to address the potential toxicity at the used concentrations of SM4 in comparison to DMSO control matching the highest concentration. For this, we have measured the % of cell viability by Trypan Blue staining and quantification. As seen in the new Figure 4 panel F, we do not see toxic effects with 25µM SM4, which nevertheless is sufficient to reduce the infection phenotype. However, at 50µM of SM4 some toxicity can be seen in the infected cells, but not in the uninfected counterparts. This indicates that the high concentration of SM4 inhibitor specifically affects viability of the KSHV-infected cells.<br><br>The 3D sprouting assays were originally carried out only with 50µM SM4 to ensure that all the cells within the spheroid would be exposed to the treatment. However, we agree that consistent treatment concentrations should be used between the different experiments. We have therefore now repeated the 3D spheroid sprouting assays with 25µM SM4, and as shown in a new Figure 4J (previously I) panel, obtained similar sprouting inhibition as with the higher, 50µM SM4, but the cells in the spheroids now do look healthier. Moreover, we performed the 3D spheroid sprouting assay with ECFCBLs, and new images are now added to Figure 4 panels J and K. Description of these new results are added also to the main text (page 16, lines 338-341).<br><br>The high specificity of SM4 for SOX18 but low cytotoxic effects at similar concentrations in other cell models have previously been reported *(Fontaine et al. 2017, PMID: 28163017) in vitro* with +50 µM and *in vivo* with 25 mg/kg/day for 10 days *(Overman et al. 2017, PMID: 28137359).*
7.	It was quite curious that a mixture of ECFLY were implanted with LEC for the xenograft experiments. Are there any markers available to distinguish ECFCLY and LEC cells?<br><br>Without a control implantation of K-ECFCLY alone, the tumorigenic potential of K-ECFCLY and the contribution of the LECs is unclear. As it stands now, this experiment does not inform in vivo tumor potential of the K-ECFCLY.	Unfortunately, there are no markers that could reliably distinguish the juvenile LEC and ECFCLY. As seen in Supplemental Figure 1 panel B, also the juvenile LECs from Promocell used in our studies express CD34 that is considered as an endothelial precursor marker.<br><br>We thank the reviewer for raising this important point and apologize that the use of LECs was not better justified in the original text. The experiment was performed and histological images in Figure 5, panel B were obtained using 100% of K-ECFCLYs. This was the first (pilot) *in vivo* experiment, and when we found that the implanted K-ECFCLYs survived in the Matrigel plug until day 30, we decided to collect them for analyses. We, however, did not see any growth of the cell plugs and therefore did not call them tumors. Yet, the observed long-term survival of cells indicated the possibility to use this model for testing the efficacy of SM4 treatment *in vivo*. This is now more clearly described in the results section (page 17, lines 347-357).<br><br>We subsequently determined if the addition of a small amount of K-LECs to the K-ECFCFLYs would contribute to the inflammatory microenvironment, by providing more spontaneously lytic cells, and

	produce more infectious viruses thereby contributing to the tumorigenic growth of the K-ECFCLYs. K-LECs do not survive long times in culture, mostly due to their highly lytic nature, and thus cannot be utilized for *in vivo* experiments as such. Therefore, 5-10 % of K-LECs was mixed with 90-95% of K-ECFCLYs in the SM4 *in vivo* experiment (Figure 5C-E). However, the engrafted mixed cell population did not lead to tumorigenic growth either but supported the KS hallmarks (spindling and genome maintenance) as well as the K-ECFCLYs alone did in the pilot experiment.
The percentage of GFP varied dramatically from 20X to 63X, it is concerning that there is a high level of tissue autofluorescence in the 20X image that might influence 'relative GFP intensity'.	The images in the original Figure 5B were acquired with a confocal microscope, and the use of different magnifications and exposures influences the intensity of GFP. The 63x image was taken with a shorter exposure for GFP to be able to see the nuclear LANA dots as the brighter GFP would otherwise mask this signal from the same cells. Due to this, the intensity of GFP shows variation from the 20x image. To avoid misunderstanding we have now removed the merge image taken with the 63x objective and added a new Supplemental Figure 5 with panel A showing the autofluorescence from implanted, uninfected ECFCLY which do not express GFP. Similarly, the autofluorescence in panel C 20x images influencing the relative GFP intensity quantified in panel D-F, has been subtracted using the signal obtained from samples of the non-infected ECFCLY plugs, which do not express GFP (shown in the new S5 Fig panel B). This is now explained in more detail in the Materials and methods section; Imaging and analysis (page 38-39, lines 798-806).
Was LANA used to confirm any of the GFP data for the xenograft analysis?	We performed LANA staining for all the samples, but its quantification to confirm the GFP data from the xenografts was not included in the manuscript. To respond to this reviewer's question, the stainings were repeated for all the sections, however, we were not able to obtain a reliable quantification. The GFP signal was analysed from whole sections imaged with a confocal microscope (20x), however, the signal from the nuclear LANA dots was acquired with a 63x objective from areas with the highest GFP signal in the whole section. Representative images of LANA staining acquired from the SM4, and Vehicle groups have now been added to the revised Figure 5 panel C.
SM4 at this concentration seems to be leading to tissue necrosis.	Yes, SM4 at this concentration indeed seems to lead to necrosis as seen in Figure 5, panel C. The necrosis in the SM4 samples is specific for infected cells, as similar necrotic areas were not found in the uninfected ECFCLY grafts from mice treated with SM4. Images of the uninfected ECFCLYs graft sections have now been added as a new Supplemental Figure 5, panel B. This in vivo data is in accordance with the *in vitro* data, where viability of only the KSHV-infected ECFCLYs was affected at the highest concentration of 50μM SM4 (now shown in the revised Figure 4, new F panel). Additionally, we have now added our observations of potential side-effects during the *in vivo* SM4 treatment to the methods section (page 37, lines 767-774).
Did the authors examine for markers of proliferation or cell death in the GFP+ infected cells compared to the uninfected cells in the tissue, with or without SM4?	We did not examine markers for proliferation or cell death from tissue sections since our focus was on the hallmarks of KS phenotype (spindling cells and genome copy numbers).

Reviewer #2:	Response
1. Figure 4I-J: The sprouting assays are described only in the context of SM4 treatment, yet they seem to be significant more generally, with respect to how KSHV infection affects ECFCs. A comparison of blood versus lymphatic ECFCs (as was done for proliferation and angiogenesis) should be done.	We have now included new 3D spheroid sprouting assays in Figure 4 with ECFCBL (panel J) and K-ECFCBL (panel K) treated with either DMSO or SM4 at concentration of 25µM, which was sufficient to diminish GFP signal and the sprouting phenotype without any toxicity in K-ECFCLYs. Similar to what we have previously reported with BECs (*Cheng et al., 2011; PMID: 22177562*), uninfected ECFCBL already showed angiogenic sprouting in 3D, which did not change by KSHV infection. Neither uninfected nor K-ECFCFBLs were affected by the SM4 treatment, further indicating specificity of the SOX18 inhibition with SM4.
2. Figure 4A-H: concentrations of up to 50 microM SM4 were used. Please confirm that the DMSO concentration in all drug preparations was at the same level as in the DMSO only control.<br><br>Also, it is important to provide a confirmation that the inhibitor is working in a SOX18-specific fashion in this system, for example by testing inhibition of SOX18-DNA binding.	We thank the reviewer for raising this important point. The original experiments included three controls: DMSO at corresponding concentrations as in the different treatment conditions (1µM, 25µM and 50µM of SM4). However, for the simplicity, only values from the control sample with highest DMSO concentration (same as used with 50µM SM4) were included. To improve the transparency of the data, we have now revised the Figure 4 G-I panels so that the DMSO control data is always shown next to its corresponding SM4 treatment.<br><br>The inhibitor SM4 was identified as the lead compound from a screen of several small molecules to have high specificity for SOX18 with relatively low cytotoxic effects *(Fontaine et al. 2017, PMID 28163017)*. SM4 has been shown to perturb both protein-protein and protein-DNA interactions of SOX18 by disrupting its homo- and heterodimerization. In KSHV infection models, we have shown in *Gramolelli et al. 2020 (PMID 32518203);* Fig4E that SM4 is effective in reducing the KSHV genome copies only in cells expressing SOX18; SM4 treatment reduced KSHV genome copies in iSLK.219 only when SOX18 was ectopically expressed, but not in the SOX18-negative controls. This supports the specificity of SM4 on SOX18 inhibition. Additional data now added to the revised Fig 1D show that K-ECFCBL do not express SOX18 (or only at very low levels), and SM4 does not affect the infection phenotype of K-ECFCFBLs (Fig 3J), in contrast to K-ECFCLYs which express high levels of SOX18. More detailed studies on SOX18-DNA binding in this system was not in the scope of our study but will be addressed in the future studies.
3. Figure 5: For the mouse model, ECFCs were mixed with LEC prior to implantation. To validate this method and the exact contribution of the admixed LEC, some in vitro work with mixed cultures should be performed.<br><br>Also, please clarify the meaning of the term ‘aberrant’ in Fig 5A.	We thank the reviewer for raising this important point and apologize that the method of mixing K-LECs to K-ECFCLYs was not better explained and justified in the original text. We have now performed additional *in vitro* experiments with a mixture of K-ECFCLY (90-95%) and K-LEC (5-10%) and the data is added as a new Supplemental Figure 4. This new figure includes the same *in vitro* studies as were carried out with 100% K-ECFCLY and 25µM SM4, i.e. microscopy (A), normalized KSHV genome copies (B), percentage of LANA+ cells (C) and KSHV titers from the virus release assay performed on naïve U2OS target cells (D). Also, a new panel E of images of spheroids formed from the mixture of K-ECFCLYs and K-LECs was added.<br><br>The word ’aberrant’ was indeed not proper in this context. Our aim was to describe strong infection (nearly 100% of cells expressing GFP and having spindle morphology), so we replaced it with ‘prominent’

	to better describe the nature of the infection. We have now changed Fig 5A to an updated version.
In Fig 5B: the difference in GFP intensity is interesting. Is this seen in vitro infection too? I assume yes but please clarify.	Is the reviewer referring to the difference in GFP signal of images taken with a 20X or 63X magnification? If yes, please see our response to the Reviewer #1 comment 7, 4th paragraph.
Are the GFP-bright cells thought to be LECs or a ECFCs?	The bright GFP cells in Figure 5, panel B are all K-ECFCLYs, as we implanted 100% K-ECFCLYs in this pilot experiment. Only in the panels C-E we used a mixture of K-ECFCLY and K-LEC. Moreover, in these grafts the cells most probably are all K-ECFCLY type since they represented 90-95% of the implanted cells and they can survive in NSG mice at least up to 30 days. K-LECs are more spontaneously lytic and do not survive long after the engraftment.
**Reviewer #3**:	**Response**
None noted	
**Part III – Minor Issues: Editorial and Data Presentation Modifications**<br>Please use this section for editorial suggestions as well as relatively minor modifications of existing data that would enhance clarity.	
**Reviewer #1**:	**Response**
Minor issues: Fig1C and Fig 2D 'ECFCLY' headers are split between two lines.	Thank you for pointing this out. This has now been corrected.
**Reviewer #2**:	**Response**
1. In the Author Summary, lines 48-50, KSHV-induced transformation of lymphatic ECFCs is described as 'minimal'. This descriptor when used in an absolute sense is accurate, but since it is being used here as a comparative term, a more appropriate term should be found. As it stands, it sounds as if the infected lymphatic ECFCs have less transformative potential than the other conditions tested, when in fact the opposite is true. Perhaps 'modest' would be a better term to use. Alternately the second part of the sentence could be revised to make the result clearer. I stress this relatively minor point because the author summary is a part of the paper that may be read first, making the need for clarity important.	Thank you for an excellent suggestion; we have now changed wording in the author summary to better describe our findings.
2. Methods and Materials: The descriptions of the isolation of ECFCs in Seattle versus Helsinki appear to be different. The distinctions/similarities between the procedures should be clarified, and any difference in the phenotype of the final cell products noted. In addition, it should be indicated in the methods which EC preps (US vs. Finland) were used for the different experiments.	We appreciate the reviewers concerns as the two different ECFC isolations were not clearly explained and it was not made completely clear which ECFC preparation (USA or FIN) was used in each experiment. We have now indicated in the methods which preparation was used for each different experiment.

	Also, please confirm (if known) that blood products were from KSHV-negative adults or indicate otherwise.	The blood products in the USA or Finland are not routinely tested for KSHV, however, KSHV seropositivity is very low in both countries. The isolated ECFCLY from four healthy donors in Finland were tested negative for KSHV DNA by qPCR using primers specific for KSHV genes LANA and K8.1.
3.	Methods and Materials: Please specify the type of tissue culture surface and growth medium used for the isolation and culture of ECFCs from the Puget Sound Blood Bank samples.	We have added more detailed information to the methods section to address this point.
4.	Methods and Materials: Infections were performed with different MOIs of KSHV. How was the amount of virus/infectious virus in the stocks determined?	wtKSHV titers were determined by infecting tert-immortalized microvascular endothelial (TIME) cells with dilutions of each virus preparation, followed by immunofluorescence staining using anti-LANA and ORF59 antibodies at 48 hours post infection. The rKSHV.219 titers were determined by infecting naïve U2OS cells using serial dilutions of the concentrated virus preparation followed by staining with anti-LANA antibody and quantification of the number of LANA+ cells 24h post-infection using automated high content microscopy. This is done for each new virus preparation and the number of LANA+ cells can be compared for standardization of the quality and infectivity of the preparations. These descriptions have now been added to the methods section (page 26-27, lines 563-566 and 574-576).
5.	Figure 1C-G (and other relevant figures): Were the samples shown from the high MOI or low MOI infection protocol?	Cells in Figure 1C-H were infected with low MOI, except for the panel G where cells were infected with adjusted high MOI to achieve similar rates of infection in the beginning of the episome maintenance comparison. This was previously only mentioned in the Materials and methods section: Viruses and infection. To clarify this important detail further, indications of MOIs are now added to the figure legends.
6.	Please provide additional comments on the testing of sub-populations of ECFCs versus (I assume bulk) ECFCs. What was the rationale for using these different isolation methods and can any useful conclusions be drawn from this comparison (from either a biological or a method optimization standpoint)?	The different isolation methods were initially done independently in two different laboratories from two different countries (US and Finland) and therefore the methods differ. However, it is very assuring that similar results were obtained with the ECFCLYs from several different donors, despite of the different isolation procedures.
7.	Regarding experiments performed under limited growth factor conditions (lines 191-196), was a comparison of blood vs. lymphatic ECFCs under limited growth factor conditions ever performed? If yes, what was the result; if not this should also be noted. Also, what is the significance of this result?	This experiment was not initially performed using ECFCBL and K-ECFCBL but has now been carried out and the results are added to the revised Figure 2, panel C. The significance is that KSHV confers better survival to the K-ECFCLY compared to the mock infected or K-ECFCBL cells under growth factor deprived conditions. Revised panel B and a new panel D have now been included in the revised Figure 2, to highlight increased proliferation and survival of K-ECFCLYs compared to K-LECs, which do not support long-term growth and cannot be used in preclinical *in vivo* studies. This further encouraged us to test the survival of K-ECFCLYs also in NSG mice to be used as a novel *in vivo* KS model.
8.	Figure 2D: To improve rigor, some quantitation should be applied to this set of experiments, including with	We have provided quantitation of branch points per field of view for this set of experiments as requested (see Fig 2 panels E & F). We have not monitored the assay for longer than 24 hours as this is

different donor samples and infections (e.g., counting of branch points and lumens). Were the cord structures monitored for longer than 24 hours post-plating? If yes, when did they start to lose stability and were the kinetics similar?	when we previously saw differences in tube formation for uninfected and infected mature endothelial cells *(DiMaio et al. 2014, PMID: 25275137)*.
9. For the gene expression analysis, since blood ECFCs are less KSHV-permissive than their lymphatic counterparts, how was the degree of infection equalized?	Tittering experiments were performed for each cell type to determine the amount of virus needed to achieve similar infection rates using immunofluorescence staining for LANA and ORF59. This has been clarified in the Methods section.
10. Discussion: please comment on the significance of the observation that KSHV infection decreases EC proliferation. For example, does this have biological significance or is it an in vitro anomaly?	Please see the response to reviewer 1, point 3. We and others have previously published that KSHV infection leads to a slight decrease in proliferation of endothelial cells but it is impossible to understand the biological significance *in vivo* versus *in vitro*. In the discussion we have pointed out that KS is a slow-growing indolent tumor so rapid cell proliferation might not be expected.
11. Typographical/grammatical corrections requested:<br>Line 67: change 'induce' to 'induces'.<br>Line 154: change 'capable to support' to 'capable of supporting'<br>Line 415: change 'EFCFLYs' to 'ECFCLYs'<br>Lines 753-756: figure legend is poorly written. Please revise.	Noted, and all corrected. The figure legend has been revised.
**Reviewer #3**:	**Response**
1. For the experiments shown in Figure 1, which compare blood and lymphatic ECFCs with BECs and LECs, ECFCBLs and/or BECs are often omitted. While the overall conclusions from these experiments are still well-justified, a more consistent presentation would be preferable.	We agree with the reviewer and have now generated new data with ECFCBLs and added them to the revised Fig1 panels C, D, E, F and H, and Fig2 panel C*,* Fig 4 panels J and K, and S1 Fig panel A and B.
2. It might be helpful to readers to include at least parts A and E of Supplemental Figure 3 as one of the main figures. At present there is no visual representation of the RNA-seq results to directly accompany the text.	Please see the response to reviewer 1, major point 5 for a response to this point.
3. The specific MOI used for the various infections is not indicated in most cases.	The low or high MOI used in each experiment is now clarified in the Materials and methods section (page 26, lines 559-562) and added to the figure legends.
4. In the "siRNA Transfections" section of the Materials and Methods, it is not clear whether the amounts given are for each well or for the whole plate.	The siRNA amounts used were clarified and now reported in the Materials and Methods (page 29, lines 611-616) as 50nM/well diluted from the Dharmacon siRNA 50µM/µl stocks.
5. It might be helpful to include catalog numbers for reagents, if possible.	The catalog numbers have now been added to most of the reagents.
6. Figure 1 part C has a labeling error where the KSHV-infected ECFCLY group is just labeled "K-".	The labels were accidently split between two rows. This has now been corrected, thank you.

7. There is a typo on line 274, where "blot" should be "plot".	Noted, and corrected.
8. There is a typo on line 444, where "suing" should be "using".	Noted, and corrected.
