# Supplementary material for: KSHV infection of endothelial precursor cells with lymphatic characteristics as a novel model for translational Kaposi’s sarcoma studies
Source: PLoS Pathog. 2023 Jan 23;19(1):e1010753. doi: 10.1371/journal.ppat.1010753 (PMC9894539; doi:10.1371/journal.ppat.1010753)
Supplement: Attachment — Submitted filename: Response to reviewers R2.docx [file ppat.1010753.s012.docx]

| **PLOS Pathogens**  **KSHV infection of endothelial precursor cells with lymphatic characteristics as a novel model for translational Kaposi’s sarcoma studies**  PPATHOGENS-D-22-01283 R2 | |
| --- | --- |
| **Part I - Summary** Please use this section to discuss strengths/weaknesses of study, novelty/significance, general execution, and scholarship. | |
| **Reviewer #1**:  The authors were very responsive to my concerns with modifications to all figures and their respective text descriptions, additional details for materials and methods, and the addition of more bioinformatic data. These changes deepen and strengthen the initial characterization of a novel lymphatic endothelial cell system in the field that supports KSHV infection and viral-driven colony formation and angiogenesis in cell culture and in immune deficient mice. This system is useful for investigating fundamental aspects of pathogenesis and for the investigation of therapeutics such as the SOX18 inhibitor SM4. | |
| **Reviewer #2**:  This manuscript describes the nature of KSHV infection in circulating endothelial colony forming cells (ECFCs) that express markers of lymphatic versus blood endothelium. Similar to primary LEC, lymphatic ECFCs, are more permissive to KSHV infection than blood ECFCs, support a degree of spontaneous lytic reactivation and form small colonies in soft agar. Importantly, lymphatic ECFCs can be used in a mouse model for in vivo KS/KSHV translational studies and here the utility of the SOX18 inhibitor SM4 is shown. This is a revised manuscript that has been significantly improved by additional method detail, new data and discussion comments. A few minor details still require attention. | |
| **Part II – Major Issues: Key Experiments Required for Acceptance** Please use this section to detail the key new experiments or modifications of existing experiments that should be absolutely required to validate study conclusions. | |
| **Reviewer #1**: | **Response** |
| None |  |
| **Reviewer #2:** | **Response** |
| Regarding the comparison of KSHV genes in K-ECFCBL and K-ECFCLY in cells maintained under standard in vitro culture conditions, the relative gene expression patterns between the two cell types were remarkably similar, with the exception of K15 (higher in K-ECFCLY). This finding is shown in supplementary figure 3D and mentioned in the associated text. The authors should revisit this finding in the discussion and comment further on its potential significance. | We thank the reviewer for pointing out the need to discuss the new Supplemental Figure 3 panel D. Further comments are now added to the text at page 22, lines 461-468 (Clean version or Track Changes with Simple Markup). |
| Figure 4 includes new data showing the effect of SM4 on K-ECFCLY and ECFCLY 3D-sprouting at 25uM as well as 50uM doses, and on K-ECFCBY and ECFCBY 3D-sprouting but only at the 25uM dose. Was a 50uM dose tested on the blood-origin cells and if so, what was the result? For example, were spheroids unaffected at the higher dose of SM4 or was non-specific toxicity observed and did mock and KSHV-infected cells yield the same phenotype. Even if actual figure panels are not added, a comment should be added to the text. | The additional data on ECFCBL and K-ECFCBL were acquired only with 25 µM of SM4 and the DMSO control.  This concentration was sufficient to reduce the KSHV-infection hallmarks in K-ECFCLY without unspecific toxicity, and hence we decided to use only this concentration with spheroids of ECFCBLs and K-ECFCBLs.  The highest SM4 concentration of 50 µM shows toxicity for the K-ECFCLYs expressing high levels of SOX18, likely sensitizing the cells for SM4 (Figure 4, panel F). 50 µM seems to cause only slight non-specific toxicity to uninfected cells, having less SOX18. As shown in Figure 1 panel D, K-ECFCBLs practically do not express SOX18 (or express at very low levels), and as SM4 is SOX18-specific inhibitor, we did not expect the treatment to have an effect on the phenotype of these cells. Alas, we do acknowledge that testing the toxicity of 50 µM on K-ECFCBLs could have further highlighted the specificity of SM4. However, the focus is on the inhibitory effects of SM4 on SOX18-positive K-ECFCLYs, and thus we did not venture on the effects of SM4 treatment to the SOX18-negative ECFCBL further.  We have now clarified this further in the manuscript text (page 16-17, lines 341-348 and page 22, line 478). |
| **Part III – Minor Issues: Editorial and Data Presentation Modifications** Please use this section for editorial suggestions as well as relatively minor modifications of existing data that would enhance clarity. | |
| **Reviewer #1**: | **Response** |
| Minor issues remain regarding Figure 4. Panels A-I were not available in the Figure 4 download but there were no major issues in the original submission. With regard to Fig 4J-K, the bright field images do not match the IFA. Please clarify if these images were taken separately due to technical constraints. | We appreciate the comment, as this should have been clarified in the figure legend; the phase contrast images in Fig 4 panels J-K indeed do not always match the confocal images due to technical constraints.  The phase contrast images (upper row) are taken from *in vitro* cultures of live spheroids (located in the middle of the wells to avoid shadows or unfocused view towards the corners of the wells) with a Nikon benchtop microscope. Then, the spheroids are fixed and scooped from wells for immunofluorescence staining, which occasionally causes some of the spheroids to break down. Due to this, the phase contrast images of the sprouting phenotype are taken prior to the staining. The confocal images of GFP/RFP in Figure 4J-K were taken from the spheroids that remained intact throughout the staining procedure. For each treatment condition, two biological replicates with three or more spheroids were imaged, all showing similar phenotype and GFP/RFP. The representative images selected for the figure were the ones with least autofluorescent debris or bubbles blocking any view on the spheroid. In some cases, we were not able to select the exact same spheroid from the images taken with two different microscopes.  We have now clarified this in the Figure 4 legend, page 42, and lines 888-890, and the description of phase-contrast microscopy in the methods (page 36, lines 747-749). |
| **Reviewer #2**: | **Response** |
| The wording in the first few sentences of the Results section discussing the permissivity of the different cell populations to KSHV infection is a bit clumsy. One suggestion is to reword the third sentence (lines 126-127) as follows: “To determine whether these lymphatic ECFCs (ECFCLYs) resemble neonatal LEC with respect to an enhanced susceptibly to KSHV-infection, we seeded both ECFCLYs and blood ECFCs (ECFCBLs) onto 6-well….”  The comment on BEC not supporting lytic infection (line 143) sounds a bit dogmatic. I suggest modifying this statement so it is less definitive, for example: “Next, we analyzed if either population of ECFCs could support spontaneous lytic replication and production of new infectious viruses, a phenotype typically observed only in KSHV-infected LECs, but not BECs”  For the text in line 185, please replace “ended up with” with another descriptor, e.g., “harbored” or “maintained”.  Line 252: please change ‘to soft agar’ to ‘in soft agar’.  Lines 314-316: please change to ‘at the protein level’ and ‘the mRNA level’.  Line 330: please change to ‘concentrations of….’.  Line 368: please change to ‘a spindling phenotype…” | We appreciate all of these good suggestions to improve the wording. We have now added all the suggested modifications to the manuscript text. |
